# Life Satisfaction Among Adolescents: Validation of the Adapted Multidimensional Students’ Life Satisfaction Scale

**DOI:** 10.3390/bs14111042

**Published:** 2024-11-05

**Authors:** Anna Tabueva, Victoria Ismatullina, Timofey Adamovich, Artem Malykh, Pavel Kolyasnikov, Sergey Malykh

**Affiliations:** Scientific Laboratory “Center for Population Research”, Ural Federal University named after the first President of Russia B.N. Yeltsin, 620062 Ekaterinburg, Russia; anntabueva@gmail.com (A.T.); victoria2686@gmail.com (V.I.); tadamovich11@gmail.com (T.A.); malykhartem86@gmail.com (A.M.); pavelkolyasnikov@gmail.com (P.K.)

**Keywords:** Multidimensional Students’ Life Satisfaction Scale, life satisfaction, adolescents, gender, psychometric analysis

## Abstract

Life satisfaction is associated with adolescents’ adaptability, academic achievement, and mental health, and it reflects the profile of a country’s economic development. In this study, we assessed the psychometric properties of the Russian version of the Multidimensional Students’ Life Satisfaction Scale (MSLSS). The initial adaptation of the MSLSS was performed using a sample of primary school students. Validation on a sample of early-to-middle adolescents is necessary to assess its reliability and validity for this age group. The sample comprised 2826 adolescents between ages 10 and 16 (Mage = 12.6, SD = 1.2, 41.3% girls) who completed the Russian version of the MSLSS assessing their perceived life satisfaction across five scales. While the five-factor structure of the MSLSS was confirmed, the psychometric analysis indicated that the statements function more effectively measured life satisfaction as a singular one-factor construct. We also observed a significant age-related decline in overall life satisfaction and satisfaction with family, self, school, friends, and teachers. Boys reported significantly lower satisfaction with school compared to girls. This study confirms the validity and reliability of the Russian version of the MSLSS, thereby augmenting its general applicability. Furthermore, we replicated previously reported age and gender differences in life satisfaction among early-to-middle adolescents.

## 1. Introduction

In recent years, subjective well-being and life satisfaction have garnered significant attention within psychological research, reflecting a growing recognition of their critical role in assessing the overall quality of life and societal progress. As economies develop, understanding these concepts provides valuable insights into the impacts of economic policies on citizens’ happiness and mental health, thereby highlighting the importance of a holistic approach to development that goes beyond mere economic indicators [1,2]. Due to such burgeoning demand, research on well-being and life satisfaction has become relevant not only for adults but also for children: studies highlight the importance of well-being for children’s overall adaptation [3,4] and mental health (e.g., [5]), with school-related well-being predicting higher adaptability and positive behavior [6,7,8,9]. Although low-achieving students do not necessarily report low well-being and high-achieving students do not necessarily report high levels of well-being, there is still a significant relationship between academic achievement and subjective well-being in children [10,11,12]. Studies also demonstrate that adolescence is a particularly challenging period for well-being: although this age stage is identified as one of the healthiest periods in life [10], research shows that well-being is most susceptible to decline at this age [13,14,15].

In 1994, Huebner E.S. developed the Multidimensional Students’ Life Satisfaction Scale (MSLSS) for students starting in grade 3 [16]. The creation of the MSLSS made it possible to assess children’s satisfaction profiles in five key areas (family, school, living environment, self, and friends) and identify their generalized life satisfaction. In addition, the MSLSS demonstrates sufficient psychometric properties and has a reliable five-factor structure, indicating the psychological validity of the scales measured [17,18,19].

In 2018, the MSLSS was first translated into Russian and adapted for students in Russian primary schools [20]. The Russian adapted version of Multidimensional Students‘ Life Satisfaction Scale allows for an assessment of life satisfaction on five subscales, family, school, teachers, self, and friends, as well as a general indicator of life satisfaction starting from grade 3 [20]. The adaptation of the scale for Russian-speaking participants required replacing the original MSLSS subscale living environment with the teachers subscale due to the cultural characteristics of Russia [20]. The authors argued that the decision to supplement the MSLSS with a scale measuring satisfaction with teacher–student relationships was made due to the importance of educational activities, especially in early childhood [20]. Following that, the living environment scale was excluded for the sake of compactness and due to its limited utility for school psychologists. Moreover, unlike in the United States, where the methodology was developed, Russia exhibits less regional variation [21,22], which affects the reflection of this sphere and its contribution to children’s quality of life. As a result, the teachers subscale is aimed at the assessment of a child’s satisfaction with relationships with teachers. The school subscale assesses children’s satisfaction with school life in terms of support for their interests and general attitude towards school and educational activities. The content of the statements in the family subscale refers to respondents‘ satisfaction with their relationships with family members. The self subscale concerns the level of satisfaction with oneself and a positive attitude towards oneself. The friends subscale is aimed at studying satisfaction with peer relationships. Validation of the Russian-adapted MSLSS on students in grades confirmed the correspondence of the empirical structure to the theoretical structure, consisting of five correlating factors with high internal consistency [18,20,23].

Currently, there is no information on the psychometric properties of the Russian MSLSS in assessing the life satisfaction of Russian middle and high school students. Validation on the sample of early-to-middle adolescents is necessary to assess the psychometric properties of the scale and confirm its reliability and validity for this age group. Furthermore, testing on a sample of middle school students will assess the quality of the translation of the scale into Russian and its compliance with modern requirements for measuring instruments. Taking into account the previously conducted validation study of the scale on a sample of elementary school students, it is necessary to clarify whether the scale is able to detect the changing aspects of school students’ life satisfaction that may vary in adolescence. 

The results of this study will be useful for the further usage of the scale in practical work with adolescents and for the development of further tools for mental health assessment in this age group. In this regard, the purpose of this study is to estimate the psychometric properties of the MSLSS scale on a Russian-speaking sample of middle school students (grades 5–8). We aimed to investigate the following research questions:

**Research Question 1:** Does the Russian MSLSS factorial structure remain invariant for a sample of early-to-middle adolescents, comprising five correlated factors?
**Research Question 2:** Does the Russian MSLSS demonstrate sufficient item functioning?
**Research Question 3:** Do the measurement results reflect the specifics of life satisfaction in early-to-middle adolescence?

## 2. Materials and Methods

### 2.1. Participants and Procedures

Given that Russia comprises eight federal districts, each representing a unique environment with varying levels of national representation, data collection was performed in one region of one of the major districts. Participants were selected using a stratified random sampling approach, which allowed to ensure representation across grades 5 to 8. The study involved 4002 students from the grades 5, 6, 7, and 8 of 142 Russian general education schools, which comprised 2% of the regional population of early-to-middle adolescents. 

The data were collected anonymously using an online platform designed specifically for testing purposes. Informed consent for participation was obtained from parents by school administrations in advance. Students completed the Multidimensional Students’ Life Satisfaction Scale as part of an online battery of questionnaires administered in the classroom during regular school hours. The entire set of assessments required approximately 45 min to complete and was presented in a randomized order. Data collection was facilitated by the teachers. The analysis of individual variability in and sequence lengths of answers revealed that 29.4% of students responded carelessly, and these results were discarded. A total of 2826 participants (1485 girls, 52.5%) were selected for further analysis (Table 1). The average age of participants was 12.6 years old (10–16 years old, SD = 1.2).

### 2.2. Measures

The adapted Russian version of The Multidimensional Students’ Life Satisfaction Scale was used [20]. The scale contains 30 statements aimed at assessing life satisfaction on five subscales: family, school, teachers, self, and friends. Item statements in English are presented in Table 2; the corresponding statements in Russian are available from [20]. Participants were asked to indicate how often each statement is true on a Likert scale from 1 (never) to 5 (always). For further analysis, responses to the reverse statements S4 and S6 were coded in reverse: “never”—5 points, “sometimes”—4 points, etc. The total score and scores by subscales were calculated by summing the ratings.

### 2.3. Statistical Analysis

The assessment of the psychometric characteristics of the Russian MSLSS was conducted within the frameworks of Classic Test Theory and Item Response Theory with R 4.3.1 software environment using the mirt [24] and lavaan [25] libraries.

We firstly conducted a reliability analysis to evaluate the internal consistency and stability of the MSLSS measures. This analysis involved estimating item-to-total Spearman’s rank correlation coefficients (*ρ*) to assess the relationship between individual items and the overall scale score. Additionally, Cronbach’s alpha (α) was calculated for each subscale to determine the internal reliability of the items, providing insight into the cohesiveness of the constructs measured.

To test our first research question regarding the invariance in the factorial structure of the MSLSS among early-to-middle adolescents, we employed a structured statistical analysis strategy. Initially, we calculated Spearman’s rank correlation coefficients (*ρ*) with False Discovery Rate (FDR) adjustment for multiple comparisons to assess the relationships among the five subscales of the MSLSS. To further evaluate the factor structure, the sample was randomly split into two subsets. Exploratory Factor Analysis (EFA) was performed on the first half of the data with promax rotation, examining a five-factor solution compared to a one-factor solution to determine the most appropriate model fit. Subsequently, the identified factor solution was tested for its stability and validity on the second half of the data using Confirmatory Factor Analysis (CFA). The fit of the factor models was evaluated using indices such as the Chi-square (χ^2^) statistic, the Tucker–Lewis Index (TLI), and the Root Mean Square Error of Approximation (RMSEA) with an emphasis on achieving acceptable thresholds for model fit.

To assess our second research question regarding the sufficient functioning of the MSLSS statements, we employed Item Response Theory (IRT) utilizing both five-factor and one-factor Graded Response Models (GRMs) for comparative analysis. The GRM was considered to be a commonly used option for the analysis of multivariate Likert-type data [26]. Model fit was evaluated using a set of fit indices, including the M2 statistic, the Root Mean Square Error of Approximation (RMSEA), the Standardized Root Mean Square Residual (SRMSR), the Tucker–Lewis Index (TLI), the Comparative Fit Index (CFI), and the Akaike Information Criterion (AIC) and Bayesian Information Criterion (BIC). Subsequent item-to-model fit was analyzed through the Orlando and Thiesen S-χ^2^ statistic. Following this, the functioning of each item and response category across the latent trait continuum was estimated as discrimination values of statements and respective threshold values of response categories. To enhance the interpretability of the results, we then visualized category probability curves and information functions for each item of the MSLSS. Finally, we graphically represented the test information function along with the standard error function to examine the scale’s precision and reliability of measurement.

To evaluate our third research question regarding whether the measurement results of the MSLSS were sensitive to the specific characteristics of life satisfaction in early-to-middle adolescence, we conducted an analysis of variance (ANOVA) to examine potential differences based on gender and grade. The two-way ANOVA was conducted with factors of 2 (gender: male vs. female) and 4 (grade: 5 vs. 6 vs. 7 vs. 8) with subsequent Tukey’s HSD post hoc comparison for both the total MSLSS score and for each of the MSLSS subscales.

## 3. Results

### 3.1. Data Distribution

**Figure 1:**
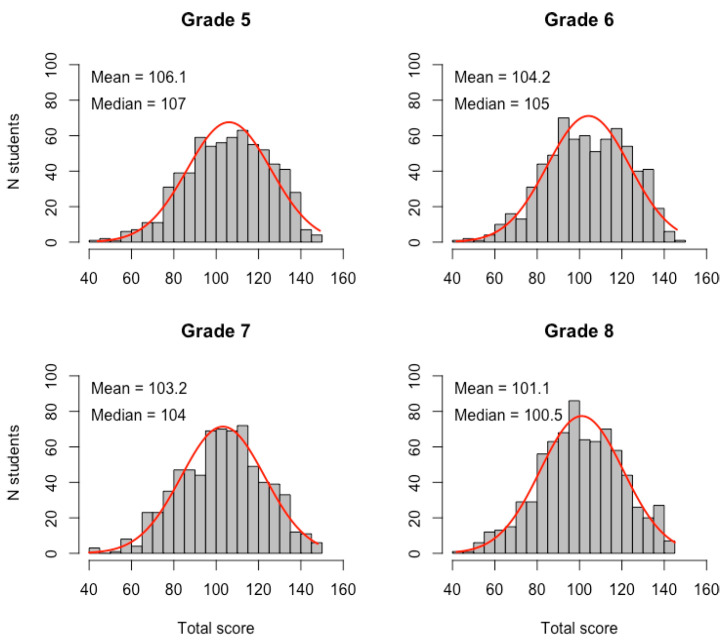
Data distribution histograms of the MSLSS total score for grades 5, 6, 7, and 8.

Table 3 presents the distribution characteristics of total scores on the five MSLSS subscales, as well as the distribution histograms of the MSLSS total score in Figure 1. The Shapiro–Wilk test was used to analyze the normality of the distribution of the total scores for each class. Due to the significant deviation from the normal distribution (*p* < 0.05), nonparametric tests were utilized for further analysis.

### 3.2. Reliability

The item-to-total correlations for the 30 MSLSS statements were calculated using Spearman’s *ρ* coefficient. The mean item-to-total correlation across all statements (Mean *ρ* = 0.6) indicated a moderate level of consistency among the statements. The statement T3 demonstrated the highest correlation, with an item-to-total correlation of 0.67, suggesting that this item is the most aligned with the overall construct of life satisfaction within the scale. Conversely, statements S4 and S6 exhibited the lowest item-to-total correlations, with values of 0.28 and 0.33, respectively. The remaining statements exhibited item-to-total correlations ranging between 0.46 and 0.67, generally reflecting acceptable-to-good levels of agreement.

To assess the internal consistency of the MSLSS subscales, Cronbach’s α was estimated for each of the five subscales. Specifically, the results indicated strong internal consistency, with values of α = 0.85 for the family subscale, α = 0.81 for the school subscale, α = 0.89 for the teachers subscale, α = 0.86 for the self subscale, and α = 0.90 for the friends subscale. The results showed that the MSLSS statements tend to measure the same underlying construct of life satisfaction consistently.

### 3.3. Factor Structure

The correlation analysis of life satisfaction scores among students in grades 5–8 revealed a significant positive relationship (*p* < 0.001) between the five subscales: family, school, teachers, self, and friends. Spearman’s *ρ* correlation coefficients, ranging from 0.27 to 0.7, indicated a moderate relationship between the measured levels of life satisfaction on the five main subscales (Table 4). The statements of the five subscales were also significantly related to each other (0.09 < *ρ* < 0.75, *p* < 0.05), with the exception of statement S4 of the school subscale, for which there was no relationship with a number of statements from the self subscale (statements SF1, SF2, SF4, and SF6), friends subscale (statements Fs3, Fs4, Fs5, and Fs6), and family subscale (statement Fa3, Fa4, and Fa6).

To examine the factor structure of the MSLSS questionnaire, the sample was randomly divided into two equal parts. The first part of the sample was used to perform an EFA to evaluate the number and composition of potential subscales. Factor extraction with promax rotation was performed for a five-factor solution, as well as a one-factor solution. The thresholds for exploratory factor model selection included a TLI greater than 0.95 in comparison to the null model, an RMSEA of less than 0.06, and the assessment of χ^2^ statistics. The results of the EFA indicated the five-factor solution as being the better fitting model (Table 5). Following the identification of the potential subscales, CFA was performed on the second part of the sample to assess the statistical viability of the five-factor solution. CFA results demonstrated acceptable goodness-of-fit for TLI, RMSEA, and χ^2^ values, supporting the exploratory analysis’ findings (Table 5). 

The analysis of the modification indices within the model of five correlated factors demonstrated that the scale items exhibited factor loadings on multiple factors and shared correlated observation errors, suggesting the potential presence of a higher-order common factor.

### 3.4. Item Functioning

For the item-functioning analysis performed on a full sample, the modeling outcomes, specifically the goodness-of-fit statistics of the one-factor and five-factor solutions, are presented in Table 6. 

It was expected that the IRT model would demonstrate results similar to CFA analysis and that the five-factor model would show better fit compared to the one-factor model. For the five-factor correlated model, the resulting RMSEA = 0.039 and SRMSR = 0.036 suggest that the data fitted the model reasonably well according to the suggested thresholds of RMSEA < 0.05 and SRMR < 0.08 [27]. The TLI = 0.959 and CFI = 0.968 were also above the recommended threshold of >0.95 [27]. Lower values of M2 statistic, RMSEA, and SRMR, as well as higher CFI and TLI values, indicated the closest fit of the model to the true data, which allowed us to favor the logistic model of five factors (Table 6). The following item fit analyses showed discrepancies between overall model fit and item-level fit across different dimensionality models. According to the Orlando and Thiesen S-χ^2^ test, which assesses the consistency of statements with the logistic model, only 20 items fit well within the preferred five-factor model, while for the one-factor model, 29 items out of 30 fit. The five-factor IRT solution demonstrated the best overall model fit, yet it suffered from poor item fit, with only 20 out of 30 items adequately functioning within the model. This discrepancy suggests that while the five-factor model captured the complexity of distinct dimensions of life satisfaction, substantial overlap among the subscales led to inadequate item functioning, evidenced by high correlations between items and subscales (Table 4) that effectively measure similar aspects of the overarching construct. Conversely, the one-factor solution, despite showing the worst overall model fit, achieved a commendable item fit with 29 out of 30 items performing well within the framework. This indicates that most items were closely aligned in their measurement of life satisfaction as a singular construct, providing consistency at the item level, even if the simplistic model failed to account for the multidimensional nature of life satisfaction. To effectively address the challenge of balancing model complexity with sound item measurement, we opted for the one-factor model for item functioning analysis. This decision was grounded in the recognition that the MSLSS is primarily employed to assess overall life satisfaction, rather than dissecting it into distinct subscales, making the simplified one-factor model applicable and practical for our purposes.

Table 7 displays the results of item fit and function analysis of the MSLSS statements within a one-factor logistic model. The non-significant *p*-value of the S-χ^2^ test suggests that 29 out of 30 items fit the model with statement Fa3, with “My family is better than most others” being the only exception (S-χ^2^ *p*-value = 0.001). The item discrimination parameter α indicates the differentiating ability of statements and is high for most statements (Mean = 1.42, SD = 0.3): six statements show low-to-moderate discrimination parameters (<1.34), three statements show very high discrimination parameters (>1.70), and for the remaining statements, the discrimination parameter is high (1.35–1.69). The teachers subscale contains questions with the highest discriminative value (Mean α = 1.61) among other subscales. The statement of the friends subscale Fs6, “My friends will help me if I need it”, and the statement of the teachers subscale T5, “I can always turn to my teachers for help”, demonstrate the highest discriminative values (α = 1.74 and α = 1.73, respectively). The school subscale exhibits the lowest average discrimination parameter for its statements (Mean α = 1.08), including statements with discrimination parameter values that are more than two standard deviations below the mean, specifically statement S4 (“I wish I didn’t have to go to school”), with α = 0.44, and statement S6 (“I feel bad at school”), with α = 0.64. 

**Figure 2:**
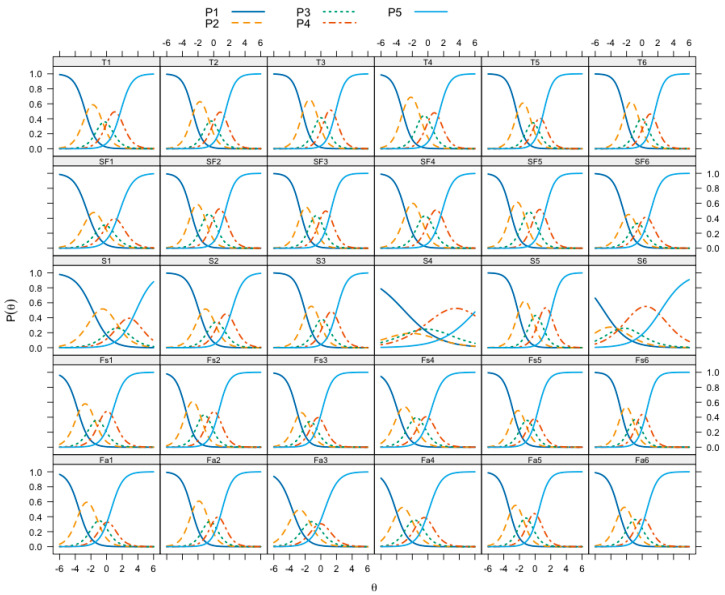
Item Response Category Curves of each MSLSS statement: θ—the level of the measured life satisfaction trait; P(θ)—the probability of endorsing a response category depending on the level of the measured trait.

The results also demonstrate that the category threshold estimates β that correspond to the level of the measured trait at which the probability of endorsing a response category or above is equal to the probability of choosing the categories below increase monotonically for each MSLSS statement (Table 7, β1, β2, β3, β4). The largest range of the measured latent trait of life satisfaction is covered by the answer categories of MLSS statements on the school subscale (from −4.95 to 6.20), while the smallest range is covered by statements on the friends subscale (from −3.90 to 0.83). The probabilities of approving response categories on a scale from never (1) to always (5) depending on the level of the measured life satisfaction trait are presented in the form of probabilistic response curves for each MSLSS statement in Figure 2. 

**Figure 3:**
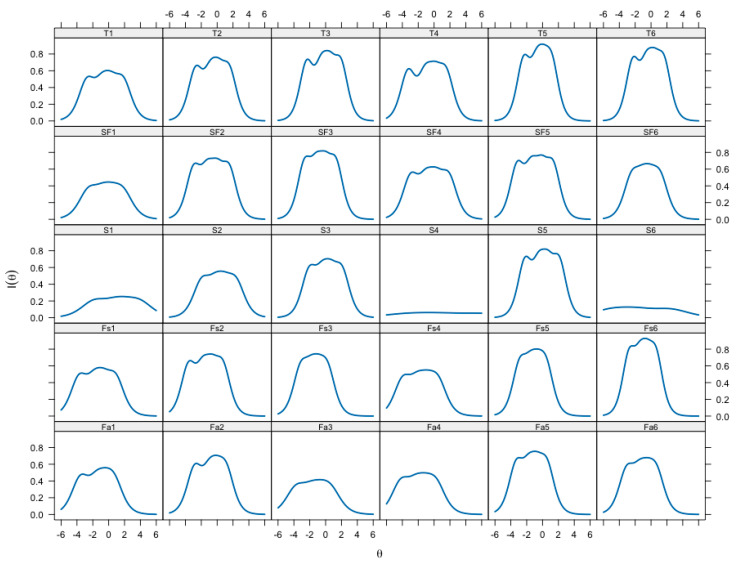
Item Information Functions of each MSLSS statement: θ—the level of the measured life satisfaction trait; I(θ)—the amount of gained test information depending on the level of the measured trait.

As the level of the measured life satisfaction θ increases, the probability of endorsing the category response P(θ) increases and then decreases as responses move to the next higher category. The results indicate that the response categories cover a wide range of the latent life satisfaction traits, especially statements S4 and S6. Moreover, for these statements, the flat location of the response category curves indicates a low differentiating ability α (Table 7, α): the probability of approving response categories for these statements changes relatively slowly when the level of the latent trait (θ) changes. In addition, for statements S1 and S4, the locations of the response category curves shift to the right, which indicates the increased “difficulty” of the statements, namely even with higher levels of life satisfaction, the probability of endorsing the response category “Never” (“Always” for the opposite statement S4) remains high. At the same time, the curves of response categories for the family and friends subscales, which shift to the left, indicate the relative ease of the statements of these subscales, that is, even with low levels of life satisfaction, the likelihood of agreement with these statements remains high. The accuracy of measurement depending on the level of life satisfaction at the level of statements is estimated with the information function (Figure 3): higher levels of information lead to more accurate estimates of the expression of the latent trait.

**Figure 4:**
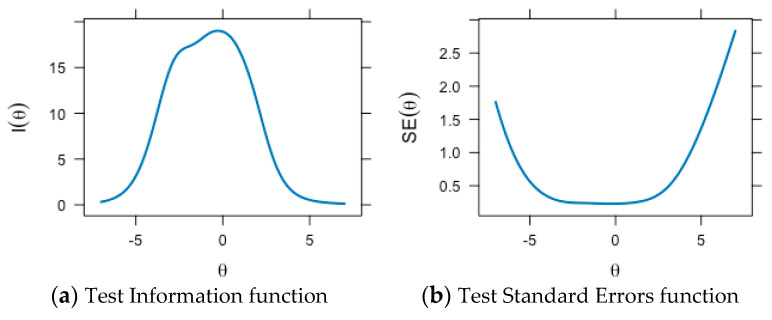
Test Information Functions and Test Standard Errors for the measurement of the MSLSS total score: (**a**) Test Information function; (**b**) Test Standard Errors function. θ—the level of the measured latent trait; I(θ)—the amount of gained test information depending on the level of the measured trait; SE(θ)—the standard error of measurement depending on the level of the measured trait.

For statements S4 and S6 of the school subscale, the slope of the information function remains close to zero across the entire spectrum of the measured life satisfaction trait, which suggests that the accuracy of the estimates is low for all levels of life satisfaction. Statement S1 is also uninformative, especially for subjects in the lower half of the trait being measured. The shift of the information functions of the statements of the family and friends subscales to the left suggests that the average levels of satisfaction are measured accurately, and lower levels are measured more precisely than the high ones. The statements providing the greatest informativeness for assessing the latent trait of life satisfaction are T5, “I can always turn to my teachers for help”, from the teachers subscale and Fs6, “My friends will help me if I need it”, from the friends subscale. The test information function summarizes the information functions of the MSLSS statements and allows us to assess the range of expression of life satisfaction, in which the MSLSS total score provides the most information (Figure 4a). 

The MSLSS total score yields the highest amount of information within the range of the measured life satisfaction level from −4 to 2. This indicates that the MSLSS scale is more precise for assessing the lower half of the latent trait’s expression, thereby allowing for a more accurate estimation of individuals who are less satisfied with their lives. Accordingly, the standard error of measurement is increased at low and high levels of life satisfaction (Figure 4b).

### 3.5. Demographic Differences

The two-way ANOVA results indicated a significant main effect of grade for total MSLSS score, F (3, 2731) = 6.69, *p* < 0.001, η2 = 0.007. Further Tukey’s post hoc tests revealed that the life satisfaction of 8th graders was significantly lower than those of 5th graders (Mean = 4.62, SD = 1.04) and 6th graders (Mean = 2.74, SD = 1.04). The total life satisfaction scores in grade 8 students were significantly lower than those of grade 5 students and grade 6 students, indicating that the level of life satisfaction decreased from grade 5 to grade 8. The main effect of gender was not significant (F (1, 2731) = 3.22, *p* = 0.073, η2 = 0.001), indicating that male and female students did not differ significantly in overall life satisfaction. The interaction between gender and grade was not significant (F (3, 2731) = 0.38, *p* = 0.77, η2 < 0.001), indicating that the level of life satisfaction in middle school students from grades 5 to 8 showed similar developmental trends in both male and female children.

To further explore age and gender differences on the MSLSS subscales, two-way ANOVA with gender and grade factors was performed for each of the five MSLSS subscales. For the family subscale, the results indicated a significant main effect of grade, (F(3, 2731) = 7.71, *p* < 0.001, η2 = 0.008), with the family life satisfaction of 8th graders being significantly lower than those of 5th graders (Mean = 1.03, SD = 0.27), 6th graders (Mean = 1.14, SD = 0.27), and 7th graders (Mean = 0.86, SD = 0.27), indicating a decrease in family satisfaction with age. No effects of gender (F (1, 2731) = 0.19, *p* = 0.66, η2 < 0.001) and interaction between gender and grade (F (3, 2731) = 0.85, *p* = 0.47, η2 < 0.001) were found, suggesting that the decline in family satisfaction in middle school students from grades 5 to 8 followed similar trends in both male and female children.

For the school subscale, the results indicated a significant main effect of gender, F (1, 2731) = 12.49, *p* < 0.001, η2 = 0.005, with female students being more satisfied with school than male students (Mean = 0.66, SD = 0.19). The results also showed a significant main effect of grade, F (3, 2731) = 7.84, *p* < 0.001, η2 = 0.008, with the school life satisfaction of 5th graders being significantly higher than those of 7th graders (Mean = 0.74, SD = 0.27) and 8th graders (Mean = 1.26, SD = 0.26), indicating changes in average school satisfaction with age. However, no significant interaction effect between gender and grade was found (F (3, 2731) = 0.84, *p* = 0.47, η2 = 0.008), indicating that gender differences did not change with age.

For the teachers subscale, ANOVA results indicated a significant main effect of grade, F (3, 2731) = 13.76, *p* < 0.001, η2 = 0.015. Tukey’s post hoc tests revealed that the 5th graders’ satisfaction with teachers was significantly higher than those of 6th graders (Mean = 1.34, SD = 0.29), 7th graders (Mean = 1.37, SD = 0.30), and 8th graders (Mean = 1.75, SD = 0.29). This indicates that grade 5 students had the highest satisfaction with teachers among middle school students. No significant effects of gender (F (1, 2731) = 3.37, *p* = 0.066, η2 = 0.001) and interaction between gender and grade (F (3, 2731) = 0.60, *p* = 0.613, η2 < 0.001) were found, suggesting that the level of satisfaction with school in middle school students had similar trajectories of decline in male and female children.

For the self subscale, no significant effects of gender (F (3, 2731) = 1.34, *p* = 0.260, η2 = 0.001), grade (F (1, 2731) = 1.14, *p* = 0.285, η2 < 0.001), and interaction between gender and grade (F (3, 2731) = 0.30, *p* = 0.823, η2 < 0.001) were found, suggesting that satisfaction with self is stable in middle school students across grades in males as well as female children. For the friends subscale, ANOVA results showed a significant main effect of gender, F (1, 2731) = 4.64, *p* = 0.031, η2 = 0.002, with female students being more satisfied with friends than male students (Mean = 0.42, SD = 0.20). No effects of grade (F (3, 2731) = 2.07, *p* = 0.10, η2 = 0.002) and interaction between gender and grade (F (3, 2731) = 0.11, *p* = 0.917, η2 < 001) were found, suggesting that the boys and girls differ in their satisfaction with friends, but this does not change with age in both male and female children.

## 4. Discussion

This study makes it possible to evaluate the psychometric properties of the MSLSS questionnaire, aimed at assessing the level of life satisfaction on five subscales: family, school, teachers, self, and friends. The reliability of the MSLSS is thoroughly assessed, yielding confident results. The item-to-total correlations indicate a strong relationship between individual items and the overall scale, suggesting that each item is contributing meaningfully to the construct being measured. Additionally, Cronbach’s α values demonstrate excellent internal consistency and further affirm the consistency of the MSLSS across subjects, highlighting its stability as a measurement tool. Collectively, these findings indicate that the MSLSS is a robust instrument for assessing life satisfaction among students and can be confidently used in future research and applied settings.

### 4.1. Factor Structure Insights

Analysis of the empirical structure of the MSLSS demonstrates compliance with the theoretical structure, which includes five correlated factors. The observed five-factor structure is in line with the first research question and corresponds to the original MSLSS questionnaire [16], as well as the first Russian-language adaptation of MSLSS for students in grades 3–4 [20]. At the same time, it is revealed that the statements of each subscale have an impact on other subscales, which may indicate the presence of a common factor. These findings are supported by the better fit of the one-factor IRT model, suggesting that MSLSS measurement of life satisfaction functions more efficiently as a singular construct of life satisfaction.

### 4.2. Quality of Item Functioning

The results of the psychometric analysis of the Russian MSLSS performed on a sample of middle school students indicate a high differentiating ability of statements and the accuracy of the resulting estimates, which corresponds to the second research question. Most of the statements are highly informative; only the statements of the “School” subscale demonstrated lower differentiating ability. In a study examining the subjective well-being of Russian students, it was found that their perceived well-being was not significantly influenced by the educational environment, suggesting that the low overall discrimination ability of the school scale may be culturally contextual [28]. Specifically, statements S4 (“I wish I didn’t have to go to school“) and S6 (“I feel bad at school“) demonstrated the lowest item functioning; however, this can be attributed to the formulations of these statements: even the students that are satisfied with their life may not like going and being at school due to unrelated reasons (e.g., comfort, schedule, meals, etc.) [29]. The observed overall low discriminability of statements on the school subscale may as well be present due to the specific relationship between satisfaction with school life and overall satisfaction. According to studies, the relationship between overall life satisfaction and satisfaction with school is less pronounced compared with the correlation between the level of satisfaction with the school and the level of satisfaction with teachers; at the same time, satisfaction with school friends and satisfaction with classmates show a strong relationship with overall life satisfaction, while these factors are weakly correlated with overall school satisfaction [30,31]. The discovered correlation range between the MSLSS subscales also confirms these trends and probably indicates that, to a greater extent, it is communicative relationships at school that support the general well-being of schoolchildren [32,33]. 

The evaluation of the psychometric properties of the MLSS at the item level indicates that though the scale effectively measures different aspects of life satisfaction, most precise estimates are acquired at lower levels of life satisfaction. Specifically, the most informative subscales, friends, family, and self, demonstrate greater accuracy in assessing lower ranges of life satisfaction compared to higher ones. The most accurate measurements and the greatest differentiation of respondents can be obtained among those who are not very satisfied with life. The internal consistency of the subscales also corresponds to the expected result and corroborates the findings from the adaptation study conducted on junior school students [20]. This suggests the robust consistency and reliability of the instrument for assessing life satisfaction across various dimensions in adolescents.

### 4.3. Demographic Specifics of Life Satisfaction

According to the third research question, the Russian MSLSS version demonstrated the capacity for capturing sociodemographic differences specific to early and middle adolescents. An analysis of life satisfaction scores among middle school students indicated that boys are less satisfied with school than girls, a result consistent with the findings from the Russian MSLSS adaptation [20]; this outcome aligns with the recognized gender specificity of well-being in Russia [34]. However, gender differences in school life satisfaction may not lead to differences in the overall level of life satisfaction between genders. Some studies on gender differences in life satisfaction among children and adolescents show that it indeed remains invariant between gender groups [35,36]. On the other hand, there is evidence of systematic gender differences in subjective well-being attributed to cross-cultural differences such as higher gender parity or the GDP of the country [37,38,39,40]. 

For students in grades 5–8, a decrease in overall life satisfaction and on each subscale was found as they grew older, with the self subscale being the only exception. The stability of satisfaction with self can be explained by the lifetime stability of self-esteem and related traits [41,42,43]. Similar results are shown in studies of European middle school students; in particular, students’ overall life satisfaction is subjected to a significant decline between the ages of 11 and 14 years [14,36,44,45]. Cross-sectional studies also demonstrate a global decline in subjective well-being among adolescents [46]. The trend of declining subjective well-being with age in most countries begins around age 10 [15,47] and is considered a cross-cultural developmental phenomenon [48] that can be attributed to alterations in cognitive or social processes [49,50,51], as well as the worsening of life conditions [52,53].

### 4.4. Limitations

The primary limitation of our study is that data were collected solely from one region within a single Russian federal district, which may constrain the generalizability of our findings to wider populations. In order to enhance the evidence of the validity and reliability of MSLSS for estimating the population of Russian adolescents, it is recommended that future studies collect data from multiple regions and federal districts across Russia to strengthen the overall conclusions drawn from the data.

## 5. Conclusions

The obtained demographic differences in the level of life satisfaction between students in grades 5 to 8 indicate the sensitivity of the MSLSS methodology to the sociocultural context of Russia, which allows us to speak about the quality of the methodology under study. The overall psychometric properties and demographic differences obtained suggest that this psychometric instrument is sufficiently sensitive, accurate, and reliable to use for middle school students. The testing of MSLSS on middle school children showed the same quality of methodology as for primary school students, which allows for its further practical use to assess the life satisfaction of early-to-middle adolescents. 

## Figures and Tables

**Table 1:** The quantitative representation of middle school students for the entire sample and each grade.

Gender	All	5th Grade	6th Grade	7th Grade	8th Grade
All, N	2826	670	693	705	758
Boys, N	1254	309	317	294	334
Girls, N	1485	350	359	378	398

**Table 2:** MSLSS statements with corresponding item IDs and subcales.

Item ID	Item in English
	Family
Fa1	I enjoy spending time with my parents
Fa2	My parents engage in interesting activities with me
Fa3	My family is better than most others
Fa4	My family members treat each other well
Fa5	My parents treat me fairly
Fa6	I can do a lot of interesting things at home
	School
S1	In the morning, I feel like going to school
S2	I enjoy being at school
S3	School is interesting
S4	I wish I did not have to go to school *
S5	I find school lessons interesting
S6	I feel bad at school *
	Teachers
T1	I like most of my teachers
T2	I am satisfied with my teachers
T3	I love listening to my teachers
T4	Some of my teachers are interesting people
T5	I can always turn to my teachers for help
T6	It is interesting to talk to my teachers
	Self
SF1	I think I look good
SF2	Others find me interesting
SF3	I am a nice person
SF4	Most people like me
SF5	There are many things I do well
SF6	I like myself as a person
	Friends
Fs1	My friends are mice to me
Fs2	My friends treat me well
Fs3	I have good friends
Fs4	I enjoy spending time with my friends
Fs5	I have enough friends
Fs6	My friends will help me if I need it

Note: * reverse statements.

**Table 3:** The distribution characteristics of scores across five MSLSS subscales.

	Mean	SD	Median	Min	Max	Skewness	Kurtosis
Total	103.56	19.68	104	40	149	−0.19	2.61
Family	22.86	5.05	23.5	6	30	−0.51	2.56
School	17.65	4.83	18	6	30	−0.03	2.83
Teachers	19.27	5.39	19	6	30	0.05	2.25
Self	20.34	5.1	21	6	30	−0.21	2.46
Friends	23.45	5.25	24	6	30	−0.73	2.86

**Table 4:** Spearman’s *ρ* correlation coefficients between the five MSLSS subscales.

	Family	School	Teachers	Self	Friends
School	0.342 ***				
Teachers	0.503 ***	0.692 ***			
Self	0.586 ***	0.354 ***	0.484 ***		
Friends	0.577 ***	0.266 ***	0.388 ***	0.627 ***	

Note: *** *p*-value < 0.0001.

**Table 5:** Goodness-of-fit coefficients of MSLSS CFA models.

	Exploratory Factor Analysis		Confirmatory Factor Analysis
	1F Solution	5F Solution	5F Solution
TLI	0.573	0.929	0.916
RMSEA	0.129	0.052	0.057
χ^2^ (df)	9870.025 (405)	1432.69 (295)	2206.415 (390)

Note: 1F—single-common-factor model; 5F—five-factor model.

**Table 6:** Goodness-of-fit coefficients for different dimensionality IRT logistic models.

	M2	df	*p*	RMSEA	SRMSR	TLI	CFI	AIC	BIC
1F	10,087.5	315	0	0.105	0.118	0.702	0.728	212,474	213,366
5F	1434.9	270	0	0.039	0.036	0.959	0.968	195,756	196,915

Note: 1F—single-common-factor model; 5F—five-factor model.

**Table 7:** Item functioning parameters of MSLSS statements: discrimination values (α), threshold values (β) of response categories, and S-χ^2^ item fit statistics.

Item ID	α	β1	β2	β3	β4	S-χ^2^ *p*-Value
			Family			
Fa1	1.34	−3.53	−1.49	−0.41	0.6	0.761
Fa2	1.51	−2.82	−0.95	−0.02	1.08	0.302
Fa3	1.15	−3.66	−1.78	−0.59	0.51	0.001
Fa4	1.27	−4.14	−2.31	−1.14	0.15	0.231
Fa5	1.58	−3.29	−1.72	−0.69	0.51	0.359
Fa6	1.49	−3.04	−1.46	−0.44	0.62	0.062
			School			
S1	0.9	−1.78	0.76	1.95	3.79	0.893
S2	1.35	−1.89	−0.2	0.87	2.31	0.359
S3	1.53	−2.03	−0.4	0.65	2.03	0.076
S4	0.44	−3.07	−1.32	0.95	6.2	0.082
S5	1.67	−2.21	−0.46	0.65	2.08	0.319
S6	0.64	−4.95	−3.18	−1.5	2.4	0.062
			Teachers			
T1	1.41	−2.71	−0.8	0.26	1.79	0.661
T2	1.59	−2.69	−0.83	0.15	1.49	0.879
T3	1.68	−2.38	−0.51	0.54	1.9	0.497
T4	1.55	−3.26	−1.09	0.13	1.48	0.964
T5	1.73	−2.37	−0.73	0.11	1.11	0.062
T6	1.71	−2.27	−0.56	0.43	1.61	0.34
			Self			
SF1	1.2	−2.49	−0.76	0.3	1.7	0.164
SF2	1.58	−2.94	−1.25	0	1.48	0.796
SF3	1.66	−2.63	−1.15	−0.03	1.28	0.22
SF4	1.45	−2.92	−1.01	0.26	1.81	0.359
SF5	1.63	−3.18	−1.42	−0.11	1.3	0.696
SF6	1.47	−2.38	−1.06	−0.12	1.09	0.231
			Friends			
Fs1	1.38	−3.69	−1.78	−0.67	0.83	0.062
Fs2	1.58	−3.58	−1.79	−0.62	0.69	0.13
Fs3	1.55	−3.1	−1.82	−0.9	0.18	0.062
Fs4	1.35	−3.9	−2.11	−0.87	0.41	0.256
Fs5	1.61	−2.84	−1.52	−0.59	0.4	0.072
Fs6	1.74	−2.74	−1.36	−0.48	0.6	0.202

## Data Availability

The datasets generated and analyzed during the current study are available from the corresponding author on reasonable request. None of the experiments were preregistered.

## Abbreviations

The following abbreviations are used in this manuscript:

MSLSS	Multidimensional Student’s Life Satisfaction Scale
EFA	Exploratory Factor Analysis
CFA	Confirmatory Factor Analysis
IRT	Item Response Theory
GRM	Graded Response Model
